# Assessing initial MRI reports for suspected CJD patients

**DOI:** 10.1007/s00415-022-11087-x

**Published:** 2022-04-01

**Authors:** Aaron Jesuthasan, Danielle Sequeira, Harpreet Hyare, Hans Odd, Peter Rudge, Tze How Mok, Akin Nihat, John Collinge, Simon Mead

**Affiliations:** 1 NHS National Prion Clinic, University College London Hospitals NHS Foundation Trust, London, UK; 2 MRC Prion Unit at UCL, Institute of Prion Diseases, Courtauld Building, 33 Cleveland Street, London, W1W 7FF UK; 3 Department of Neuroradiology, National Hospital for Neurology and Neurosurgery, Queen Square, Holborn, London, WC1N 3BG UK

**Keywords:** Creutzfeldt-Jakob disease, CJD, MRI, Prion

## Abstract

**Background:**

MRI is invaluable for the pre-mortem diagnosis of sporadic Creutzfeldt-Jakob disease (sCJD), demonstrating characteristic diffusion abnormalities. Previous work showed these changes were often not reported (low sensitivity), leading to eventual diagnosis at a more advanced state. Here, we reviewed the situation a decade later, on the presumption of improved access and awareness over time.

**Methods:**

We reviewed initial MRI scans of 102 consecutive suspected sCJD patients recruited to the National Prion Monitoring Cohort study between 2015 and 2019, assessing for characteristic signal changes in the striatum, thalamus and cortical ribbon. We compared our findings to formal reports from referring centres. Requesting indications were studied to assess if they were suggestive of CJD. Patients were examined and their MRC Prion Disease Rating Scale scores recorded.

**Results:**

We identified characteristic MRI abnormalities in 101 cases (99% sensitivity), whilst referring centres reported changes in 70 cases (69% sensitivity), which was a significant improvement in reporting sensitivity from 2012. Reporting sensitivity was associated with signal change in the cerebral cortex, and with the number of regions involved, but not significantly affected by clinical information on request forms, or referring centres being regional neuroscience/non-neuroscience centres. Similar to a previous study, patients with missed abnormalities on initial reporting possessed lower MRC Scale scores when referred to the NPC than those correctly identified.

**Conclusions:**

Whilst local MRI reporting of sCJD has improved with time, characteristic abnormalities remain significantly under detected on initial scans. Sensitivity is better when the cerebral cortex and multiple regions are involved. We re-emphasize the utility of MRI and encourage further efforts to improve awareness and sensitivity in the assessment of patients with rapidly progressive dementia.

**Supplementary Information:**

The online version contains supplementary material available at 10.1007/s00415-022-11087-x.

## Introduction

Creutzfeldt-Jakob disease (CJD) is a rapidly progressive and universally fatal neurodegenerative disease, caused by the propagation of assemblies of misfolded prion protein [1]. Sporadic CJD (sCJD) remains the most common human prion disease, contributing 85% of CJD patients. Although a definitive diagnosis of sCJD relies on histopathological confirmation, clinical assessment and investigations can make near certain pre-mortem diagnoses [2, 3]. Of the investigations included in the current diagnostic criteria, diffusion-weighted MRI (DWI) is considered an essential investigation given its ready availability, short acquisition time, and high sensitivity (up to 98%) and specificity (nearly 100%) [4–8]. Characteristic features of sCJD on DWI and fluid attenuated inversion recovery (FLAIR) sequences include high intensity signal changes in cortical, striatal and thalamic regions [9, 10].

Early identification of sCJD offers opportunities for improved care, avoidance of unnecessary tests and empirical treatments, planning for end of life, and inclusion into clinical trials [6]. PRN100, a monoclonal antibody treatment recently offered to a small number of patients with sCJD, exemplifies one such innovative opportunity which relies on early patient diagnoses. Other experimental compounds are also being developed [11].

Unfortunately, diagnostic changes of sCJD on MRI are often missed, as evidenced by our paper published in 2012, where we found an overall 47% sensitivity on initial radiology reporting [12]. Subsequent efforts by the National Prion Clinic (NPC) and others to improve awareness of CJD-specific MRI abnormalities has included delivery of lectures to hospital specialists and presentation of our findings at national conferences. The inclusion of DWI to dementia protocols has also become well established. We aimed to re-explore whether there has been any change in the sensitivity of detecting sCJD on MRI, hypothesising that increased awareness amongst clinicians, improved access to MRI and scan protocols, and improved scan quality since our previous study, might have improved rates of diagnosis.

## Materials and methods

### Patients

We retrospectively studied MRI scans from patients referred to the NPC with suspected sCJD between 2/4/2015 and 5/12/2019 who were enrolled into the National Prion Monitoring Cohort study [13]. This study was approved by the Scotland A Research Ethics Committee. Informed consent was obtained from participants or, when appropriate, relatives and carers. Patients who did not fulfil the clinical diagnostic criteria for sCJD or did not possess MRI imaging and reports that were transferred and archived at University College London Hospital were excluded [2].

### MRI evaluation and reporting

The earliest MRI scan of each patient was assessed by one of three research team members (one senior neuroradiologist and two clinical research fellows, specifically trained for the purposes of the study) at the National Hospital for Neurology and Neurosurgery (NHNN) and NPC. The research team were aware of a probable sCJD diagnosis. DWI b1000 and FLAIR sequences were assessed for three types of high intensity signal change, which are characteristic of CJD: in the striatum (sufficient even if only one of caudate or putamen were affected), thalamus, or at least two cortical regions (temporal, parietal, occipital), being aware of regions that tend to give non-specific signal changes [3, 14]. It should be emphasised that thalamic signal changes alone are not included in the current sCJD diagnostic criteria. Apparent diffusion coefficient (ADC) maps were available for all patients, which were additionally used for MRI evaluation. Ambiguous findings were reviewed by all three members of the research team and a consensus agreed.

External reports from referring centres were reviewed to assess whether they included CJD as a potential diagnosis. Clinical indications within the request section of radiological reports were also reviewed to determine if they were suggestive of a diagnosis of CJD, searching for key words such as “CJD”, “prion”, “dementia”, and “cognitive decline”. The Medical Research Council Prion Disease Rating Scale (MRC Scale, a functionally-oriented 20-point outcome measure designed for patients with CJD) scores at initial assessment were recorded [13].

### Statistical analysis

Comparison of groups were made using McNemar’s test (for paired comparisons), *χ*^2^ test, or logistic regression (STATA 16.0). A *p*-value < 0.05 was deemed significant.

## Results

We obtained MRI scans and neuroradiological reports of 106 consecutive patients (56 males, 50 females). DWI sequences were available for 104 of these patients, including four patients who were later diagnosed with inherited prion disease (2 E200K, 2 P102L) following *PRNP* sequencing, whose clinical picture at the time of referral was indistinguishable from sCJD. Characteristics of the remaining 102 suspected sCJD cases (98% with DWI and 2% solely possessing FLAIR), including ultimate diagnosis, are summarised in Table 1, noting that “probable sCJD” is known to have an extremely high correlation with post-mortem diagnosis. We identified CJD-associated MRI changes in 101 of the 102 sCJD cases (99% sensitivity), with the remaining scan having subtle cortical ribboning uncorrelatable with its ADC map, which was deemed insufficient to meet diagnostic criteria and was subsequently excluded from our further analysis. In contrast, referring centres reported changes in 70 cases (69% sensitivity, *p* < 0.0001 using McNemar’s test). Of the 4 patients who were ultimately diagnosed with inherited prion disease, 3 possessed scans that had been reported to have features of prion disease by both the NPC and referring centres.

**Table 1:** Characteristics of the 102 suspected sCJD cases when referred to the NPC

	N (% of recorded)										
Final diagnosis	Definite sCJD						Probable sCJD				
	20						82				
Gender	Male						Female				
	56						46				
Average age (range)	66 (33–86)										
Average MRC scale score (range)	11 (0–20)										
Codon 129 genotype	MM			MV		VV			Not tested		
	28 (35)			31 (39)		21 (26)			22		
EEG findings	PSWCs	General slowing			Non-specific abnormalities			Normal			Unrecorded
	9	50			29			10			32
RT-QuIC	Positive	Negative			Uninterpretable			Insufficient CSF			No CSF
	84 (90)	9 (10)			1			2			6
Region of referring centre	Greater London (28), South East England (12), South West England (12), East England (10), East Midlands (3), West Midlands (6), Yorkshire and the Humber (7), North East England (6), North West England (9), Scotland (4), Wales (2), Northern Ireland (3)										
Number of cases with MRI changes (% sensitivity)	Year		Total (*n*)				NPC review			Referring centre	
	2012		91				83 (91%)			43 (47%)*	
	2020		102				101 (99%)**			70 (69%)	

A comparison of the number of cases with CJD-associated MRI changes reported by the NPC and referring centres in 2012 and 2020 is also included

*PSWC* periodic sharp wave complexes

^*^p < 0.01 compares the referring centre's opinion of CJD-associated changes in 2020 with 2012 using *χ*^2^ with Yates’ correction and 1 degree of freedom

^**^p < 0.0001 compares the referring centre’s opinion of CJD-associated changes in 2020 with the NPC opinion in 2020 using McNemar’s test

Of the sCJD cases that were reported as showing features of prion disease by referring centres, 46 had MRI requests that were suggestive of sCJD with 14 specifically mentioning CJD, whilst 19 requests were not suggestive of sCJD and 5 requests were unavailable to us. For the cases in which a differential diagnosis of sCJD was not reported, 18 cases had suggestive requests with 7 specifically mentioning CJD, whilst 9 requests were not suggestive, and 4 requests were unavailable. Suggestive clinical information within the request was not found to have a significant impact on the sensitivity of reporting by referring centres (*p* = 0.7 using logistic regression).

Our data were compared to the findings reported in 2012 (Table 1), showing a significant improvement in referring centre identification of CJD-associated MRI signal changes (*p* < 0.01 using *χ*^2^ test).

Of the 101 sCJD cases, we found that 67% of cases had restricted diffusion in the cortex, 80% had restricted diffusion in the basal ganglia and 51% had restriction of the thalamus. No MRI scans displayed thalamic signal change alone. We reviewed whether sensitivity of reporting by referring centres varied by the location of signal change (Supplementary Table 1). We fitted logistic regression models to test whether the regions of signal change (cortex, striatum, thalamus), or a simple sum of the number of sites affected (sum of sites), determined sensitivity of reporting. Both signal change within the cortex (OR 2.50, CI 1.01–6.18, *p* = 0.048) and sum of sites (OR 2.61, 1.28–5.33, *p* = 0.0083) positively predicted a report of CJD.

We also compared reporting sensitivity between referring centres that were known to be regional neuroscience centres against non-neuroscience centres. 62 patients were referred to the NPC by regional neuroscience centres, of which 45 cases were correctly reported on initial MRI scan whilst 17 possessed changes that were missed. 39 patients were referred by non-neuroscience centres, of which 25 cases were correctly reported and 14 cases were missed diagnoses. No significant difference in reporting sensitivity could subsequently be found between regional neuroscience and non-neuroscience referring centres (*p* = 0.22 using logistic regression).

Where CJD-associated MRI abnormalities were detected by the referring hospital, patients had a mean MRC score of 11.5/20 (range 0–20) on initial assessment by the NPC whilst patients with missed MRI abnormalities possessed a lower mean score of 9.7/20 (range 0–19, *p* = 0.07 using logistic regression), similar to our previous report [12]. Additionally, days since symptom onset did not predict an accurate test report (*p* = 0.44 using logistic regression).

## Discussion

**Fig. 1:**
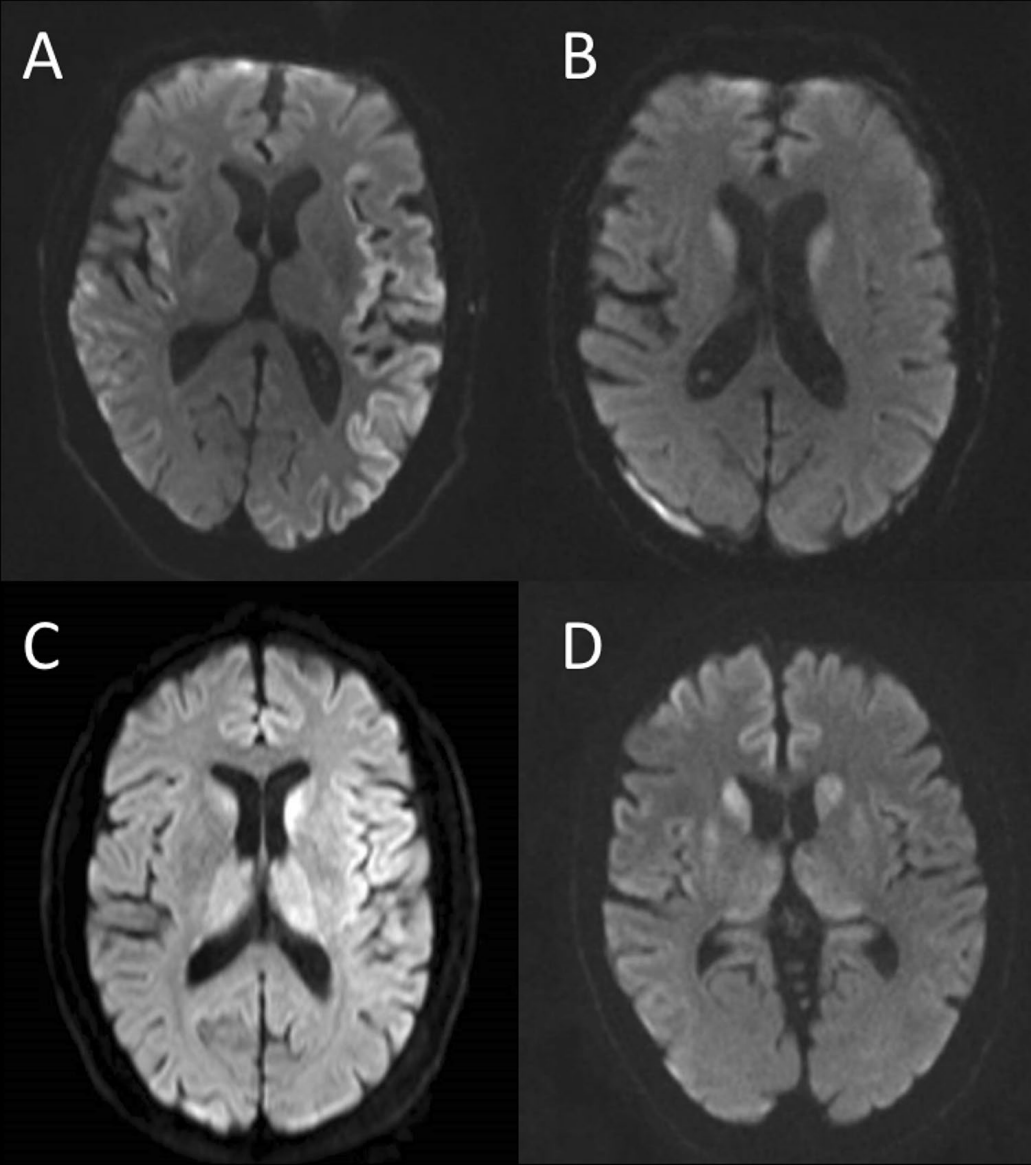
Initial MRI scans from four separate cases, not reported by referring centres as being suspicious for CJD. Image **A** displays significant cortical involvement which was noted by the radiologist, with differentials of seizure or hypoxia listed. Image **B** shows high intensity change in the striatum, which was not documented on the report. Images **C** and **D** demonstrate high intensity changes in all three regions that are characteristically affected in CJD (cortex, thalamus and striatum). These changes were missed in the report for Image **C**, whilst the report for image **D** mentioned abnormal signal in the basal ganglia only, and suggested differentials including hypoglycaemia, hypoxia, mitochondrial and metabolic causes

In this study, we found MRI changes had not been identified by referring centres in 30% of patients with sCJD, despite well-defined imaging diagnostic criteria that have a sensitivity greater than other, more invasive diagnostic tests (Fig. 1) [3, 14]. Although identification of sCJD-related diffusion abnormalities has improved since our study in 2012, these findings suggest that for a considerable number of patients, diagnosis, support and stratification for future clinical trials may be delayed [12].

Several possible reasons may explain the difference in reporting of sCJD by referring centres when compared to our reporting. Firstly, we were aware of the probable diagnosis of sCJD prior to reviewing the imaging, whereas referring centre radiologists were not provided with an imaging request suggestive of sCJD in 26% of the unreported cases. Although our results indicate the clinical information on the imaging request form does not significantly influence reporting sensitivity, our analysis did not take into account the grade of signal hyperintensity in the reported and missed groups, and it may have been that a relevant clinical indication improves reporting sensitivity in cases with subtle MRI findings [15]. As a separate consideration, any addenda to the initial MRI report following multidisciplinary meetings, may not have been updated on the actual MRI report available to us, contributing to an underestimated referring centre reporting sensitivity.

Our analysis looking at the impact of location of signal change on the sensitivity of reporting revealed that this did play a significant role on missed diagnoses, with referring centres generally picking up CJD more reliably if the cerebral cortex was involved (in isolation) and additionally as the number of involved sites increased. In those cases where a diagnosis of sCJD was missed, it may have been that high intensity signal changes were dismissed as artefact, particularly in poor-quality scans. However, DWI sequences were available in virtually all included cases, which when combined with the ADC map, should aid a radiologist to discriminate between real and artefactual signal change [9].

Similar to our study undertaken in 2012, we found that patients with MRI findings that are missed on initial scan, are referred to the NPC at a more advanced stage of disease, as demonstrated by a lower MRC Scale score on initial assessment [12], although on this occasion the finding did not achieve statistical significance. This emphasises the importance of prompt identification of CJD on MRI to allow an earlier window for specialist clinical care and recruitment into clinical trials.

The improved sensitivity of reporting is encouraging. This may, in part, be driven by an increased awareness of CJD over the years and increased utility of DWI sequences, performed in 98% of cases here compared to 79% in the previous study, potentially indicating radiologists were better provided with scan modalities to identify signal changes [5, 6, 9, 14]. The use of DWI sequences with higher b-values (e.g. *b* = 3000 s/mm^2^), correlating to the applied diffusion weighting, is also encouraged to further enhance sensitivity to CJD-associated changes [16]. Moreover, computed aided diagnosis (CAD) systems are being studied to facilitate early, accurate diagnoses of neurological disease on MR imaging, which if developed and validated for CJD, could significantly improve imaging interpretation [17].

It is important, however, to acknowledge that in certain patients, particularly those with encephalopathies who require extensive sedation for scanning to be undertaken, MRI may not be the most appropriate investigation and less invasive tests such as CSF analysis may alternatively be required. We also acknowledge that our review was not blinded to suspected diagnosis, meaning we had a very high prior suspicion of CJD related scan abnormalities, which may bias sensitivity. The balance between sensitivity and specificity in overall accuracy of MRI reporting necessitates further investigation. We could not reliably investigate this aspect because CJD mimic conditions are often identified prior to recruitment into our Cohort study.

## Conclusion

MRI is undoubtedly an investigation of great utility for the diagnosis of CJD, particularly due to its ready accessibility, however the characteristic high intensity signal changes are still often not correctly linked to CJD on initial scan reports. Encouragingly, there has been significant progress in radiological detection since 2012, although further efforts are still required to improve these figures. It is subsequently anticipated that, with further increased alertness of sCJD and its diagnostic criteria, improvements in MRI accessibility, scan quality and potentially the use of CAD, there could be significant positive strides toward earlier identification and effective management of sCJD patients.

## Supplementary Information

Below is the link to the electronic supplementary material.

Supplementary file1 (DOCX 15 KB)

## Data Availability

The datasets supporting the conclusions of this article are included within the article and its additional files.

## Abbreviations

MRI: Magnetic resonance imaging
sCJD: Sporadic Creutzfeldt-Jakob disease
NPC: National prion clinic
DWI: Diffusion weighted imaging
FLAIR: Fluid attenuated inversion recovery
NHNN: National hospital for neurology and neurosurgery
MRC Scale: Medical research council prion disease rating scale
ADC: Apparent diffusion coefficient
CAD: Computer aided diagnosis
