# Supplementary material for: Assessing initial MRI reports for suspected CJD patients
Source: J Neurol. 2022 Apr 1;269(8):4452–8. doi: 10.1007/s00415-022-11087-x (PMC9293800; doi:10.1007/s00415-022-11087-x)
Supplement: Supplementary file 1 — Supplementary file1 (DOCX 15 KB) [file 415_2022_11087_MOESM1_ESM.docx]

|  | Referring Centre Reporting | | |
| --- | --- | --- | --- |
| Brain regions affected | **Reported CJD** | **Did not report CJD** | **Sensitivity** |
| Cortex only | 11 | 7 | 61.1% |
| Striatum only | 1 | 4 | 20% |
| Cortex with thalamus | 2 | 0 | 100% |
| Cortex with striatum | 20 | 6 | 76.9% |
| Striatum with thalamus | 17 | 11 | 60.7% |
| Cortex, Striatum and Thalamus | 19 | 3 | 86% |
| Total | 70 | 31 | 69.3% |

*Supplementary Table 1: Sub-analyses of identified and missed sCJD cases from reporting of initial scans by referring centres, based on location of high intensity signal change.*
